# High Glucose Induces Lipid Accumulation via 25-Hydroxycholesterol DNA-CpG Methylation

**DOI:** 10.1016/j.isci.2020.101102

**Published:** 2020-04-29

**Authors:** Yaping Wang, Lanming Chen, William M. Pandak, Douglas Heuman, Phillip B. Hylemon, Shunlin Ren

**Affiliations:** 1 College of Food Science and Technology, Shanghai Ocean University, Shanghai 201306, China; 2 Department of Internal Medicine, Virginia Commonwealth University/McGuire VA Medical Centre, Research 151, 1201 Broad Rock Boulevard, Richmond, VA 23249, USA

**Keywords:** Biological Sciences, Molecular Genetics, Systems Biology

## Abstract

This work investigates the relationship between high-glucose (HG) culture, CpG methylation of genes involved in cell signaling pathways, and the regulation of carbohydrate and lipid metabolism in hepatocytes. The results indicate that HG leads to an increase in nuclear 25-hydroxycholesterol (25HC), which specifically activates DNA methyltransferase-1 (DNMT1), and regulates gene expression involved in intracellular lipid metabolism. The results show significant increases in ^5m^CpG levels in at least 2,225 genes involved in 57 signaling pathways. The hypermethylated genes directly involved in carbohydrate and lipid metabolism are of PI3K, cAMP, insulin, insulin secretion, diabetic, and NAFLD signaling pathways. The studies indicate a close relationship between the increase in nuclear 25HC levels and activation of DNMT1, which may regulate lipid metabolism via DNA CpG methylation. Our results indicate an epigenetic regulation of hepatic cell metabolism that has relevance to some common diseases such as non-alcoholic fatty liver disease and metabolic syndrome.

## Highlights

• High glucose induces lipids accumulation via epigenetic regulation
• High glucose induces lipids accumulation via DNA CpG methylation in promoter regions
• High glucose induces lipid accumulation by inhibiting multiple signaling pathways
• 25-Hydroxycholesterol is an endogenous agonist of DNMT1, an epigenetic regulator

## Graphical Abstract

**Figure:**
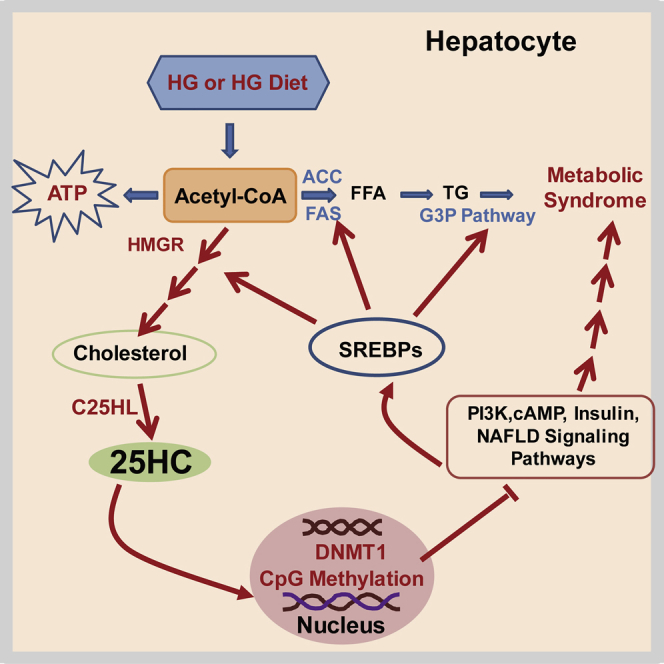

## Introduction

A high-glucose (HG) diet has been associated with metabolic syndrome (Moreno-Fernandez et al., 2018, Zhao et al., 2016), non-alcoholic fatty liver disease (NAFLD) (Liu et al., 2018), type II diabetes (Menegazzo et al., 2015, Vallon and Thomson, 2017), and cardiovascular disease (Che et al., 2018). A HG diet can induce fatty acid accumulation in liver, elevate insulin resistance index, cause oxidative stress, and increase body weight. This has led to the association of a HG diet with the development of the metabolic syndrome (Du et al., 2010). Hepatocytes play an important role in metabolizing carbohydrates and lipids in the body. Their function changes depending on the circulating levels of insulin and the sensitivity of the insulin receptor. The inability of insulin to suppress hepatic glucose production is a key defect found in metabolic syndrome (Balland et al., 2019, O-Sullivan et al., 2015). The role of insulin has been well documented. Hepatocytes respond to elevated glucose levels by packaging it into glycogen. When carbohydrates are in excess, hepatocytes can convert this excess energy into lipids. When glucose levels drop and insulin production falls, the shortage of insulin in the blood signals the hepatocytes to utilize their storage by hydrolyzing glycogen and lipids. Dysregulation of this process can result in lipid accumulation in hepatocytes, leading to the development of metabolic disorders, including NAFLD and type II diabetes (Saltiel, 2016). HG in culture media induces lipid accumulation in hepatocytes, which has been used as an *in vitro* model for the study of NAFLD (Swaminathan et al., 2013). The detailed biochemical mechanisms that allow for HG to produce lipid accumulation are not fully understood.

Epigenetic modification plays a major role in the interpretation of genetic information. Methylation at position 5 of cytosine (5-methylcytosine, ^5m^C) in DNA is an important epigenetic modification that regulates gene expression and other functions of the genome (Moore et al., 2013). Cytosine methylation in CpG is inversely correlated with transcriptional activity of associated genes as it causes chromatin condensation and thus gene silencing (Yu et al., 2014). Recently published literature has demonstrated that dysregulation of gene expression is important in metabolism and can affect tissue function and in turn the metabolic state (Castellano-Castillo and Moreno-Indias, 2019). It has been shown that HG levels affected gene expression through DNA methylation (Wang et al., 2018). However, the molecular mechanisms by which HG levels lead to DNA methylation, the changes of gene expression patterns, and lipid accumulation in human hepatocytes are not well understood.

Oxysterols are oxidized products of cholesterol. They are short-lived but can be potent biologically active molecules involved in a plethora of functions including lipid metabolism and inflammatory responses (Cyster et al., 2014, McDonald and Russell, 2010). Oxysterols have long been known for their important role in cholesterol homeostasis, where they are involved in both transcriptional and posttranscriptional mechanisms in controlling cholesterol levels (Luu et al., 2016). Recent research with oxysterols has demonstrated their novel and sometimes surprising role associated with a wide variety of cellular functions (Vurusaner et al., 2016). They act as ligands for a growing list of receptors, including some that are of importance to the immune system and lipid metabolism. Oxysterols have also been implicated in several diseases, such as NAFLD and other metabolic syndromes. However, until now their specific binding sites and mechanism of action remain unclear. The objective of this study is to investigate the role of a specific oxysterol (25-hydroxycholesterol [25HC]) in the epigenetic regulation associated with HG induction of lipid accumulation in human hepatocytes.

## Results

### HG Induces Intracellular Lipid Accumulation by Increasing Gene Expression Involved in Lipid Biosynthesis in Hepatocytes

**Figure 1:**
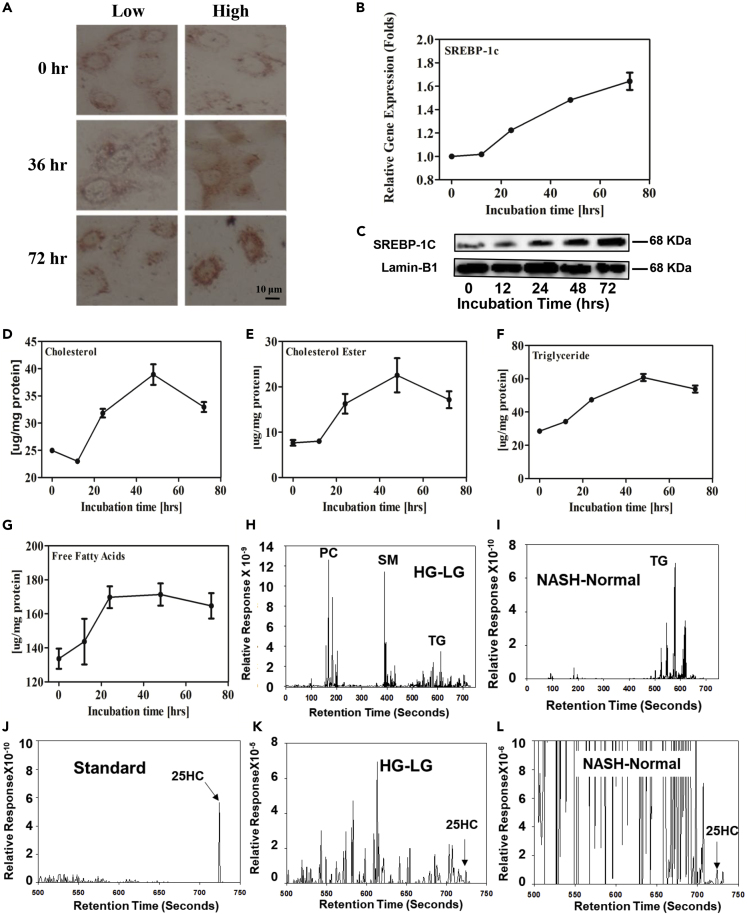
The Effects of HG on Lipid Accumulation in Hepatocytes (A–L)Huh-7 cells were treated in HG media for 0, 12, 24, 48, and 72 h. The total intracellular neutral lipids were stained with 0.2% oil red O (A); inserts are shown at 400× magnification of the boxed areas (scale bar, 10 μm); mRNA levels of SREBP-1C in HG-cultured hepatocytes were determined by real-time RT-PCR analysis (B). SREBP-1C mature form protein levels in the nuclear fraction were analyzed by western blot (C); total lipids were extracted, and individual lipids were biochemically analyzed. Hepatic total cholesterol (D), cholesterol ester (E), triglyceride (F), and free fatty acid (G). Each individual level was normalized by protein concentration. Nuclear lipid composition from hepatocytes and human liver tissues was determined by lipidomic analysis. The lipid profile in nuclear fraction from Huh-7 cells cultured in HG or LG media (HG minus LG) (H); between human normal and NASH liver tissues (NASH minus normal) (I); 25HC as standard (J); the different levels of nuclear 25HC in Huh-7 cells cultured in HG and LG (K); the different levels of nuclear 25HC in human normal and NASH liver tissue (L). The lipidomic profile represents results from one of two experiments. All values are expressed as the mean ± SD.

To study the effect of HG on intracellular lipid accumulation, Huh-7 cells were cultured in media with HG for different time periods and total neutral lipids were estimated by oil red O staining. As shown in Figure 1A, lipid levels started to increase at 36 h and had significantly accumulated at 72 h. HG increases expression and activation of SREBP-1C. Biosynthesis of free fatty acids and triglycerides is regulated by SREBP-1C (Horton et al., 2003). To investigate how HG increases lipid biosynthesis, total RNA was isolated from the HG-cultured Huh-7 cells. The mRNA levels of SREBP-1C were determined by real time RT-PCR. HG increased RNA levels in a time-dependent manner (Figure 1B). To confirm the results, SREBP-1C proteins were analyzed by western blot. As expected, increases in SREBP-1C protein levels (p < 0.01) in nuclear fractions were time dependent (Figure 1C). Thus, HG increases intracellular lipid levels by increasing SREBP-1C expression, which is consistent with previous reports (Pang et al., 2018, Xu et al., 2013).

Quantitative analysis showed that HG culture increased intracellular total cholesterol by 1.5-fold (p < 0.05) (Figure 1D), cholesterol ester by 2.9-fold (p < 0.05) (Figure 1E), triglycerides by 2.1-fold (p < 0.01) (Figure 1F), and free fatty acids by 28% (p < 0.01) at 72 h (Figure 1G). It is noted that HG culture did not significantly change free cholesterol levels (total cholesterol minus cholesterol ester). To further study the increased lipids in nuclear fractions, their composition was quantified by lipidomic analysis as shown in Figures 1H–1L and Table S2. Compared with low glucose (LG), HG increased phosphatidylcholine, sphingomyelin, and triglyceride levels with saturated fatty acids in nuclear fractions of Huh-7 cells (Figure 1H and Table S3). Compared with normal human liver tissues, non-alcoholic steatohepatitis (NASH) liver nuclear fraction showed increases mainly in triglycerides with saturated fatty acids (Figure 1I and Table S4). Interestingly, HG increased nuclear 25HC levels, whereas no change was detectable in LG (Figure 1K and Table S3); NASH liver nuclear fraction showed increases in 25HC levels by 30%, whereas decreases in 27-hydroxycholesterol (27HC) by 90% (Figure 1L and Table S4). The results imply that 25HC might play a role in the lipid accumulation.

### HG Increases Nuclear Levels of Newly Synthesized Cholesterol, 25-Hydroxycholesterol, and 27-Hydroxycholesterol

**Figure 2:**
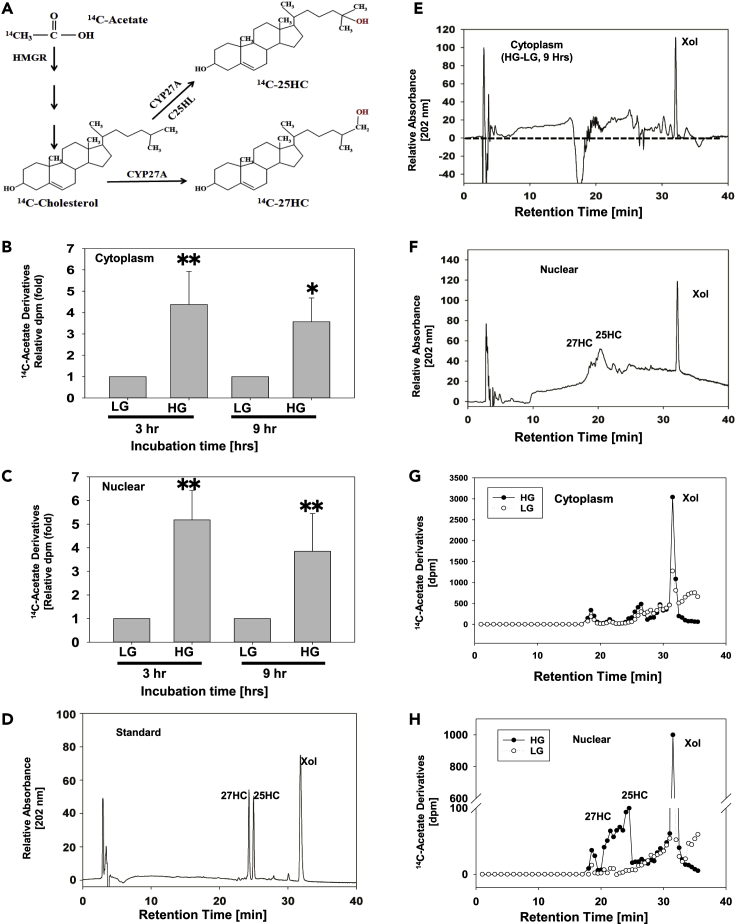
HPLC Analysis of 25HC Levels in HG-Treated Human Hepatocytes (A–H) Huh-7 cells were cultured in HG or LG media for 72 h and with [^14^C]-acetate for another 3 or 9 h. Nuclear and cytoplasmic fractions were isolated as described in Transparent Methods, partitioned into the chloroform phase, and analyzed by HPLC as described in Transparent Methods. The biosynthesis pathway of [^14^C]-sterols in the cells (A). The distribution of [^14^C]-acetate derivatives in cytoplasmic fraction (B) and nuclear fraction (C). The values represent means ± SD. HPLC standard elution profile of 25HC, 27HC, and cholesterol (D); elution profile of cytoplasmic extraction (E); elution profile of nuclear extraction (F); [^14^C]-acetate derivative profile of cytoplasmic extraction (G); and [^14^C]-acetate profile of HPLC products of nuclear extraction (H). The HPLC profiles represent one of three experiments. Data are represented as mean ± SD and statistic by t test. ∗Significant difference in distribution between LG and HG (p < 0.05); ∗∗p < 0.01.

Oxysterols play an important role in lipid metabolism (Figure 2A). To examine the nuclear oxysterol levels in hepatocytes, Huh-7 cells were cultured in DMEM media with HG for 72 h. [^14^C]-Acetate was added to the media, and the cells were cultured for another 3 and 9 h (Figures 2B and 2C). Total lipids were extracted and partially purified. Individual [^14^C]-acetate derivatives were analyzed by high-performance liquid chromatography (HPLC). HPLC analysis showed that the product has the same retention time as 25HC and 27HC in our HPLC system (Figures 2D–2F), suggesting that the nuclear oxysterol derivative is sulfonated 25HC and 27HC. Compared with cells cultured in LG, the cells cultured in HG exhibited significantly increased levels of [^14^C]-cholesterol and [^14^C]-oxysterols, including [^14^C]-25HC and [^14^C]-27HC in nuclear fractions. About 50% of the total [^14^C]-25HC was detected in the nuclear fraction (Figures 2G and 2H). We also studied the distribution of exogenous ^3^H-25HC and found that similar percentile of the ^3^H-25HC will go to nuclei in 1 h (data not shown). As oxysterols have been reported to increase SREBP-1C expression and 25HC is one of the most potent regulatory oxysterols (Spann and Glass, 2013, Xu et al., 2010), cholesterol, 25HC, and 27HC were selected for further study to determine which molecule serves as an epigenetic regulator and plays a key role in lipid accumulation.

### 25HC Serves as an Agonist of DNMT1

**Figure 3:**
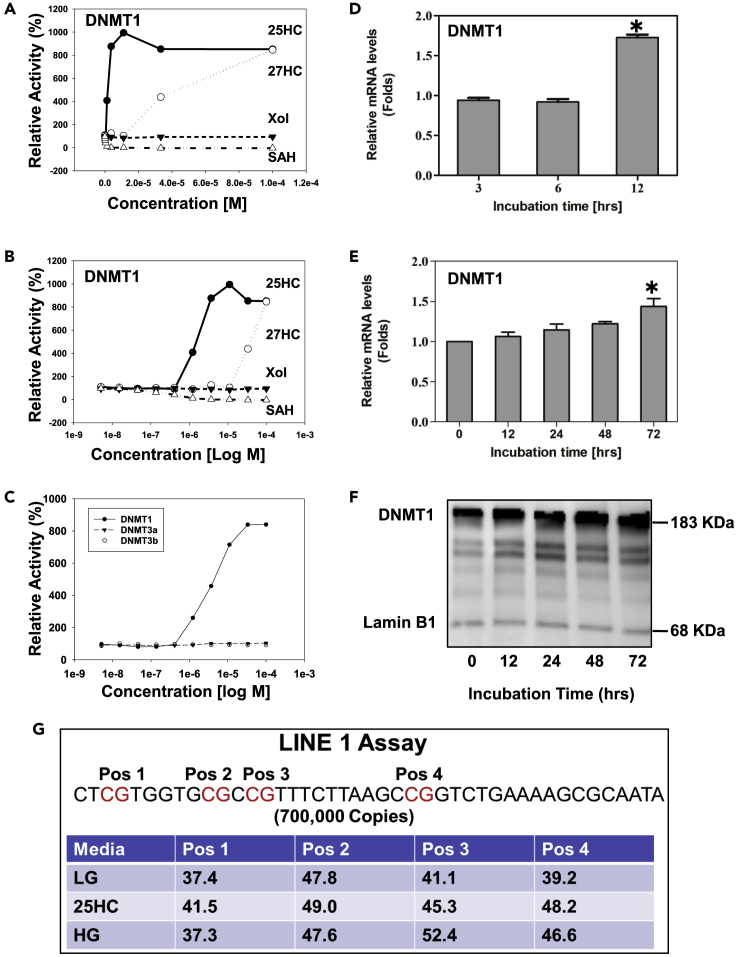
Effects of HG and 25HC on DNMT1 Expression and Enzyme Kinetics (A–G) Effects of 25HC on DNMT1/3a/3b activities were determined by enzyme kinetics. The effects of different concentration on the enzymatic activities were tested from 0 to 0.001 M (A); effects of cholesterol, 25HC, 27HC, and S-adenosyl homocysteine (SAH, inhibitor as a negative control) on the DNMT1 activity (plots versus linear) (B); and plots versus logarithm (C). Effects of HG and 25HC on the expression of DNMT1 were determined by real-time RT-PCR. Huh-7 cells were cultured in HG media for 0, 12, 24, 48, and 72 h; mRNA levels of DNMT1 were determined by real-time RT-PCR analysis (D). Huh-7 cells were treated with 25 μM 25HC for 3, 6, and 12 h. The mRNA levels of DNMT1 were determined by real-time RT-PCR analysis (E). Protein levels of DNMT1 in the nuclei were analyzed by western blot (F). Effects of HG and 25HC on the global methylation (G). After Huh-7 cells were cultured in HG for 72 h, or treated with 25 μM of 25HC for 4 h, total DNA was purified as described in Methods. The levels of methylation were estimated by LINE-1 assay. Four CpG sites in promoter region of LINE-1 element were chosen as the target position. Data are represented as mean ± SD and statistic by t test. ∗Significant difference in expression between LG and HG (p < 0.05).

Epigenetic regulators are DNA methyltransferases/demethylases and acetyltransferases/deacetylases. To study the effects of nuclear sterols on epigenetic regulation, 12 recombinant epigenetic regulators, DNA methyltransferase-1 (DNMT1), DNMT3a, DNMT3b, GCN3 (Giant congenital nevi), p300 (histone acetyltransferase), Pcaf (KAT2B lysine acetyltransferase 2B), HDAC1 (histone deacetylase 1), HDAC2 (histone deacetylase 2), HDAC3 (histone deacetylase 3), HDAC6 (histone deacetylase 6), HDAC10 (histone deacetylase 10), and KDM6B-JMJD3 (lysine demethylase 6B), were used to determine whether 25HC or cholesterol serves as their endogenous ligand(s). Interestingly, 25HC and 27HC, but not cholesterol, act on only DNMT1, and not on DNMT3a/3b (Figures 3A and 3B) and did not affect any demethylases, acetyltransferases, or deacetylases (data not shown). The kinetic study showed that 25HC increased DNMT1 by 8-fold at 3.7 μM, whereas 27HC reached the level at 100 μM, which is not physiologically significant, although both are in a dose-dependent manner. Then negative control S-adenosyl homocysteine (SAH) at 1 μM inhibited DNMT1 activity by 90% as shown in Figure 3C. Cholesterol did not have any effect on the DNMTs. The results indicate that 25HC is much more potent than 27HC (~30-fold). It is reasonable to believe that 25HC, but not 27HC, is an endogenous ligand of DNMT1, which can methylate DNA CpG and subsequently regulate gene expression. In addition, HG also increased expression of DNMT1 in hepatocytes. The RT-PCR and western blot results showed that HG and 25HC led to a time-dependent increase of DNMT1 both in mRNA and protein levels as shown in Figures 3D–3F.

Recent studies have shown that global DNA methylation (long interspersed nucleotide element 1 [LINE-1] assay) and the methylation of specific genes are related to adipogenesis (Malodobra-Mazur et al., 2019), lipid metabolism (Chen et al., 2019, Li et al., 2020), and inflammation in visceral adipose tissues (Barajas-Olmos et al., 2018, Castellano-Castillo et al., 2018), which are in turn related to the etiology of metabolic syndrome. To study the effects of 25HC on DNA cytosine methylation in the promoter region, LINE-1 analysis was first performed to estimate CpG methylation in promoter regions. Methylation usually occurs in repetitive elements, such as LINE elements. There are 500,000 LINE elements and 40 trillion copies in total. Human LINE-1 is a retrotransposable region (promoter region) and has only 700,000 copies, which correlates to ~17% of the human genome (Lander et al., 2001). The specific sequence includes four CpG dinucleotides (Pos 1, 2, 3, and 4) as methylation targets in LINE-1 (Figure 3G). The results showed that HG significantly increases CpG methylation of LINE-1 elements in Huh-7 cells. Following culture in HG for 72 h, increases in methylation of 11% at Pos 3, and 8% at Pos 4, were observed. The results indicate that HG increases CpG methylation in promoter regions.

### Profiles of Whole Genome-wide DNA Methylation in HG- and LG-Treated Huh-7 Cells

To understand the possible function of cytosine methylation in HG-treated Huh-7 cells, the cells were harvested for the construction of bisulfite-treated genomic DNA libraries. In total whole-genome bisulfite sequencing (WGBS) generated 401 million (LG) and 359 million (HG) raw reads from the two libraries by paired-end sequencing, respectively. Of the 394 million clean reads from LG library, 77% (305 million) were uniquely mapped to the reference genome of “human reference genome (hg38),” whereas of 354 million clean reads from HG library, 79% (279 million) were uniquely mapped to the reference genome, exhibiting an average read depth of 22 and 20, respectively. More than 82% cytosines were covered by at least 10 reads in the reference genome. The depth and density of the sequencing were enough for a high-quality genome-wide methylation analysis. Meanwhile, the bisulfite conversion efficiencies represented by the lambda DNA added into the libraries were over 99%, providing reliable and accurate results for the WGBS (Table S5)

**Figure 4:**
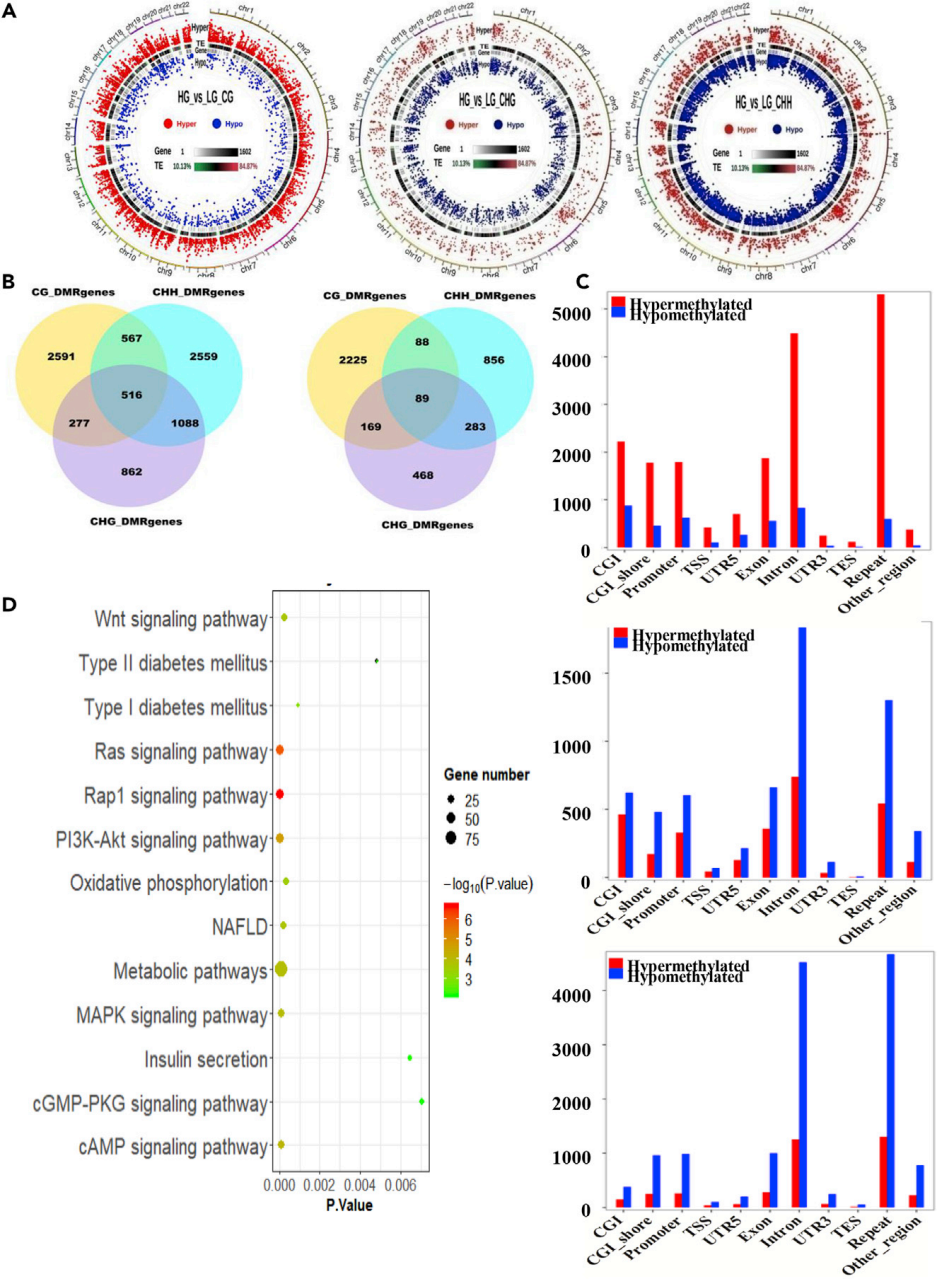
Whole-Genome Bisulfite Sequencing (WGBS) Analysis of the Effects of HG on the DNA Methylation in Hepatocytes (A–D) Huh-7 cells were cultured in HG or LG media for 72 h. Detailed global methylation was measured by WGBS. Circos maps of DMR distribution in chromosomes (A): the first circle shows the distribution of hypermethylation DMRs, the second circle shows transposable element (TE) density, and the third circle shows the distribution of hypomethylation DMRs. Venn diagrams of DMR-associated genes (DMGs) among LG and HG libraries under CG, CHG, and CHH contexts of whole genome and promoter region (B). DNA methylation levels in different genomic functional regions of the whole genome (C), where the x axis represents the different genomic regions (CGI, CGI-shore, promoter, UTR 5, exon, intron, UTR 3 and repeat) and the y axis represents the methylation levels for LG and HG libraries under CG, CHG, and CHH contexts. Significant enrichment KEGG pathways of DMGs (D).

DNA methylation levels in whole genome and differentially methylated regions (DMRs) are shown in Figure 4A. To present the global DNA methylation profiles of the two libraries, the uneven methylation levels throughout the chromosomes under CG, CHG (H represents adenosine or thymidine), and CHH contexts are shown in Figure 4B. A total of 6,012 differentially methylated genes (DMGs) were screened out among two libraries; 2,591 DMGs were identified under CG context, 862 under CHG context, and 2,559 under CHH context. In these DMGs, 277 were identified under CG and CHG contexts, 567 under CG and CHH contexts, 1,088 under CHG and CHH contexts, and only 516 under CG, CHG, and CHH contexts. Furthermore 3,549 were identified as promoter region different, and 2,225 were identified under CG context, 468 under CHG context, and 856 under CHH context. For these DMGs, 169 were identified under CG and CHG contexts, 88 under CG and CHG contexts, 283 under CHG and CHH contexts, and only 89 identified under CG, CHG, and CHH contexts.

DNA methylation levels generally show a varied distribution across different functional regions of the genome. The methylation levels in the CGI (CG island), CGI-shore (up to 2 kbp away from the CGI), promoter (upstream 2-kb sequence from transcription starting site), 5′ UTR, exon, intron, 3′ UTR, and repeat were significantly different between HG- and LG-treated groups. It is interesting that HG has significantly higher hypermethylation levels than LG in CpG positions only (Figure 4C). In total 60,633 DMRs were identified; 4,711 (2,832 hypermethylated and 1,879 hypomethylated) were distributed in CGI, 4,986 (2,727 hypermethylated and 2,259 hypomethylated) in CGI-shore, 5,105 (2,724 hypermethylated and 2,381 hypomethylated) in exon, 15,291 (7,198 hypermethylatedand and 8,093 hypomethylated) in intron, 5,312 (2,754 hypermethylated and 2,558 hypomethylated) in promoter, 21,838 (13,023 hypermethylated and 8,815 hypomethylated) in repeat region, 210 (131 hypermethylated and 79 hypomethylated) in transcription end site elements, 811 (529 hypermethylated and 282 hypomethylated) in transcription start site (TSS) elements, 757 (350 hypermethylated and 407 hypomethylated) in 3′ UTRs, and 1,612 (916 hypermethylated and 696 hypomethylated) in 5′ UTRs. In almost all DMRs, CpGs are significantly more hypermethylated than hypomethylated. It has been reported that CG methylation in promotor regions plays a key role in silencing gene expression (Baylin, 2005).

Total DMGs, 6,012 genes, were significantly enriched in 288 Kyoto Encyclopedia of Genes and Genomes (KEGG) pathways (189 hypermethylated and 129 hypomethylated). DMGs in promoter regions were significantly enriched in 169 KEGG pathways (83 hypermethylated and 51 hypomethylated) (Table S5), of which 79 (57 hypermethylated and 5 hypomethylated) were under CG context. Seventy-two DMGs (25 hypermethylated and 34 hypomethylated) were significantly enriched under CHG context and 18 (1 hypermethylated and 12 hypomethylated) under CHH context.

To analyze the function of DMGs in promoter regions under CG context, 79 KEGG pathways (p < 0.05) were identified. In these pathways 57 were identified as hypermethylation and 5 as hypomethylation. Thirteen of them are related to metabolic syndrome (Figure 4D). Six of these pathways including PI3K-Akt signaling pathway (Table 1), cAMP signaling pathway (Table 2), NAFLD (Table 3), type II diabetes mellitus (Table 4), and insulin secretion (Table 5) are directly related to carbohydrate, lipid, and energy metabolism. The most significant genes involved in these signaling pathways include insulin (INS), retinoid X receptor alpha (RXRα), insulin receptor substrate 1 (IRS1), insulin receptor (INSR), phosphoinositide-3-kinase regulatory subunit 5 (PIK3R5), mitogen-activated protein kinase 1 (MAPK1), cyclin-dependent kinase inhibitor 1B (CDKN1B), protein kinase N2 (PKN2), calcium/calmodulin-dependent protein kinase II beta (CAMK2B), peroxisome proliferator-activated receptor alpha (PPARα), mitogen-activated protein kinase 1 (MAPK1), potassium calcium-activated channel subfamily N member 3 (KCNN3), glucagon-like peptide 1 receptor (GLP1R), potassium calcium-activated channel subfamily U member 1 (KCNU1), potassium calcium-activated channel subfamily N member 4 (KCNN4), and calcium voltage-gated channel subunit alpha1D (CACNA1D). It has been well believed that insulin resistance is the major mechanism in the development and progression of NAFLD/NASH (Engin, 2017). Interestingly, DMRs in the promoter regions of key genes in these signaling pathways are hypermethylated, indicating that the gene expressions are down-regulated, which fits well with the mechanism of NAFLD mechanism.

**Table 1:** Methylation of DMR in Gene Promoter Regions of PI3K-Akt Signaling Pathway (p = 0.000523)

Gene Name	DMR Location in Promoter Region			DMR (Methylation %)		
	Chromosome	Start	End	HG	LG	HG-LG
COL11A1	Chr1	1.03 × 10^8^	1.03 × 10^8^	92.12	51.66	40.46
GNG5P2	ChrX	1.1 × 10^8^	1.1 × 10^8^	47.26	6.76	40.5
KRAS	Chr12	25250819	25250967	17.42	5.25	12.18
INS	Chr11	2162594	2162810	79.13	23.5	55.62
JAK1	Chr1	64967244	64967529	67.24	15.6	51.65
LAMA5	Chr20	62368047	62368299	31.14	8.64	22.5
BCL2	Chr18	63320785	63321134	63.22	35.11	28.1
HRAS	Chr11	536242	537214	61.26	18.46	42.8
FGFR3	Chr4	1792656	1792887	60.41	18.3	42.11
CD19	Chr16	28935890	28936133	88.21	47.13	41.08
LAMB1	Chr7	1.08 × 10^8^	1.08 × 10^8^	71.14	24.24	46.9
RAC1P2	Chr4	46724260	46724573	73.99	47.16	26.82
MAPK1	Chr22	21867333	21867621	23.71	3.56	20.15
CDKN1B	Chr12	12714273	12715037	93.4	23.71	69.69
PIK3R5	Chr17	8888466	8888776	89.6	36.73	52.87
BCL2L11	Chr2	1.11 × 10^8^	1.11 × 10^8^	72.75	42.65	30.1
PDGFB	Chr22	39242292	39242477	83.66	54.37	29.29
ANGPT1	Chr8	1.07 × 10^8^	1.07 × 10^8^	46.11	5.16	40.96
IL6	Chr7	22726101	22726775	80.53	30.36	50.18
MDM2	Chr12	68806524	68806756	75.71	33.96	41.74
INSR	Chr19	7295041	7295182	57.84	14.92	42.92
FGF11	Chr17	7437822	7438331	50.31	19.51	30.81
LPAR3	Chr1	84894412	84894734	90.41	45.1	45.31
FGF12	Chr3	1.93 × 10^8^	1.93 × 10^8^	85.93	40.52	45.41
FGFR2	Chr10	1.22 × 10^8^	1.22 × 10^8^	24.32	8.01	16.3
COL2A1	Chr12	48004804	48005182	83.53	12.91	70.62
FGF7P3	Chr9	40106680	40106914	32.35	7.07	25.28
CSF3	Chr17	40014561	40014935	73.05	18.59	54.46
FOXO3	Chr6	1.09 × 10^8^	1.09 × 10^8^	25.28	4.11	21.17
NR4A1	Chr12	52051741	52052037	61.95	14.22	47.73
F2R	Chr5	76715992	76716157	27.58	4.89	22.69
PKN2	Chr1	88684561	88684789	37.22	4.97	32.25
EIF4E1B	Chr5	1.77 × 10^8^	1.77 × 10^8^	42.38	11.45	30.93
RXRα	Chr9	1.34 × 10^8^	1.34 × 10^8^	36.99	6.91	30.08
AC113189.4	Chr17	7437822	7438331	50.31	19.51	30.81
CSF3R	Chr1	36482185	36482512	90.94	41.71	49.23
IRS1	Chr2	2.27 × 10^8^	2.27 × 10^8^	34.01	5.95	28.06
PDGFA	Chr7	521434	521719	83.23	59.88	23.35

**Table 2:** Methylation of DMR in Gene Promoter Regions of cAMP Signaling Pathway (p = 0.001699)

Gene Name	DMR Location in Promoter Region			DMR (Methylation %)		
	Chromosome	Start	End	HG	LG	HG-LG
PDE4C	Chr19	18220846	18221310	85.7	24.91	60.79
PPARα	Chr22	46149487	46149878	94.42	59.47	34.95
HCAR2	Chr12	1.23 × 10^8^	1.23 × 10^8^	87.48	45.91	41.57
GNAS	Chr20	58891431	58891578	35.11	5.71	29.4
CACNA1D	Chr3	53493469	53494019	74.84	25.81	49.03
CAMK2B	Chr7	44221409	44221698	86.07	45	41.07
RAC1P2	Chr4	46724260	46724573	73.99	47.16	26.82
PLD2	Chr17	4806842	4806937	18.7	5.64	13.06
MAPK1	Chr22	21867333	21867621	23.71	3.56	20.15
PIK3R5	Chr17	8888466	8888776	89.6	36.73	52.87
GLP1R	Chr6	39048762	39048955	86.25	59.95	26.3
ADCY1	Chr7	45574683	45574877	59.55	33.76	25.79
PDE4B	Chr1	66331798	66332539	86.73	37.91	48.82
NFATC1	Chr18	79399054	79399517	78.35	33.06	45.29
PDE4D	Chr5	59215742	59215941	94.05	54.39	39.66
VAV3	Chr1	1.08 × 10^8^	1.08 × 10^8^	88.49	24.15	64.34
F2R	Chr5	76715992	76716157	27.58	4.89	22.69
ADCY4	Chr14	24334404	24334719	86.49	53.89	32.6
HCN2	Chr19	587907	588099	56.37	20.45	35.92
GIPR	Chr19	45669075	45669744	83.03	39.41	43.62
HTR4	Chr5	1.49 × 10^8^	1.49 × 10^8^	95.66	78.7	16.96
AL590635.1	Chr6	91816380	91819294	93.85	28.92	64.92
GRIN1	Chr9	1.37 × 10^8^	1.37 × 10^8^	37.91	13.55	24.36
PPP1R1B	Chr17	39630003	39630266	65.33	17.07	48.25
NPR1	Chr1	1.54 × 10^8^	1.54 × 10^8^	76.11	53.96	22.15

**Table 3:** Methylation of DMR in Gene Promoter Regions of Non-alcoholic Fatty Liver Disease (NAFLD) (p = 0.003206)

Gene Name	DMR Location in Promoter Regions			DMR (Methylation %)		
	Chromosome	Start	End	HG	LG	HG-LG
RAC1P2	Chr4	46724260	46724573	73.99	47.16	26.82
MTCYBP22	Chr5	1 × 10^8^	1 × 10^8^	90.01	52.51	37.50
RXRα	Chr9	1.34 × 10^8^	1.34 × 10^8^	36.99	6.91	30.08
MTCO3P22	Chr5	1 × 10^8^	1 × 10^8^	88.87	45.71	43.16
MTCO1P46	Chr2	2.12 × 10^8^	2.12 × 10^8^	83.83	26.28	57.54
MTCO2P22	Chr5	1 × 10^8^	1 × 10^8^	88.87	45.71	43.16
INSR	Chr19	7295041	7295182	57.84	14.92	42.92
AC234779.1	Chrx	1.41 × 10^8^	1.41 × 10^8^	89.36	19.05	70.31
COX6CP2	Chr20	50478286	50478554	81.66	43.62	38.04
BID	Chr22	17774417	17774536	20.06	5.89	14.17
MAP3K5	Chr6	1.37 × 10^8^	1.37 × 10^8^	81.00	40.19	40.81
PIK3R5	Chr17	8888466	8888776	89.60	36.73	52.87
BCL2L11	Chr2	1.11 × 10^8^	1.11 × 10^8^	72.75	42.65	30.10
COX6B2	Chr19	55354472	55354665	85.45	65.5	19.95
PPARα	Chr22	46149487	46149878	94.42	59.47	34.95
INS	Chr11	2162594	2162810	79.13	23.5	55.62
IL6	Chr7	22726101	22726775	80.53	30.36	50.18
IRS1	Chr2	2.27 × 10^8^	2.27 × 10^8^	34.01	5.95	28.06
DDIT3	Chr12	57521695	57521932	68.03	14.01	54.02
NDUFA4L2	Chr12	57240882	57241348	72.33	37.01	35.32

**Table 4:** Methylation of DMR in Gene Promoter Regions of Type II Diabetes Mellitus (p = 0.032118)

Gene Name	DMR Location in Promoter Regions			DMR (Methylation %)		
	Chromosome	Start	End	HG	LG	HG-LG
INSR	Chr19	7295041	7295182	57.84	14.92	42.92
INS	Chr11	2162594	2162810	79.13	23.5	55.62
SOCS2	Chr12	93572989	93573585	87.30	53.22	34.08
MAPK1	Chr22	21867333	21867621	23.71	3.56	20.15
HK1	Chr10	69318270	69318400	63.47	43.17	20.30
PIK3R5	Chr17	8888466	8888776	89.60	36.73	52.87
CACNA1D	Chr3	53493469	53494019	74.84	25.81	49.03
IRS1	Chr2	2.27 × 10^8^	2.27 × 10^8^	34.01	5.95	28.06

**Table 5:** Methylation of DMR in Gene Promoter Regions of Insulin Secretion (p = 0.035573)

Gene Name	DMR Location in Promoter Regions			DMR (Methylation %)		
	Chromosome	Start	End	HG	LG	HG-LG
ADCY4	Chr14	24334404	24334719	86.49	53.89	32.6
INS	Chr11	2162594	2162810	79.13	23.5	55.62
KCNN3	Chr1	1.55 × 10^8^	1.55 × 10^8^	83.22	46.68	36.54
CHRM3	Chr1	2.4 × 10^8^	2.4 × 10^8^	58.07	21.85	36.22
GLP1R	Chr6	39048762	39048955	86.25	59.95	26.3
KCNU1	Chr8	36928346	36930601	64.67	11.97	52.7
KCNN4	Chr19	43774123	43774347	31.44	10.69	20.76
GNAS	Chr20	58888560	58888756	41.48	18.28	23.2
CACNA1D	Chr3	53493469	53494019	74.84	25.81	49.03
ADCY1	Chr7	45574683	45574877	59.55	33.76	25.79
CAMK2B	Chr7	44221409	44221698	86.07	45	41.07

### Target Next-Generation Bisulfite Sequencing to Confirm the WGBS Results

**Figure 5:**
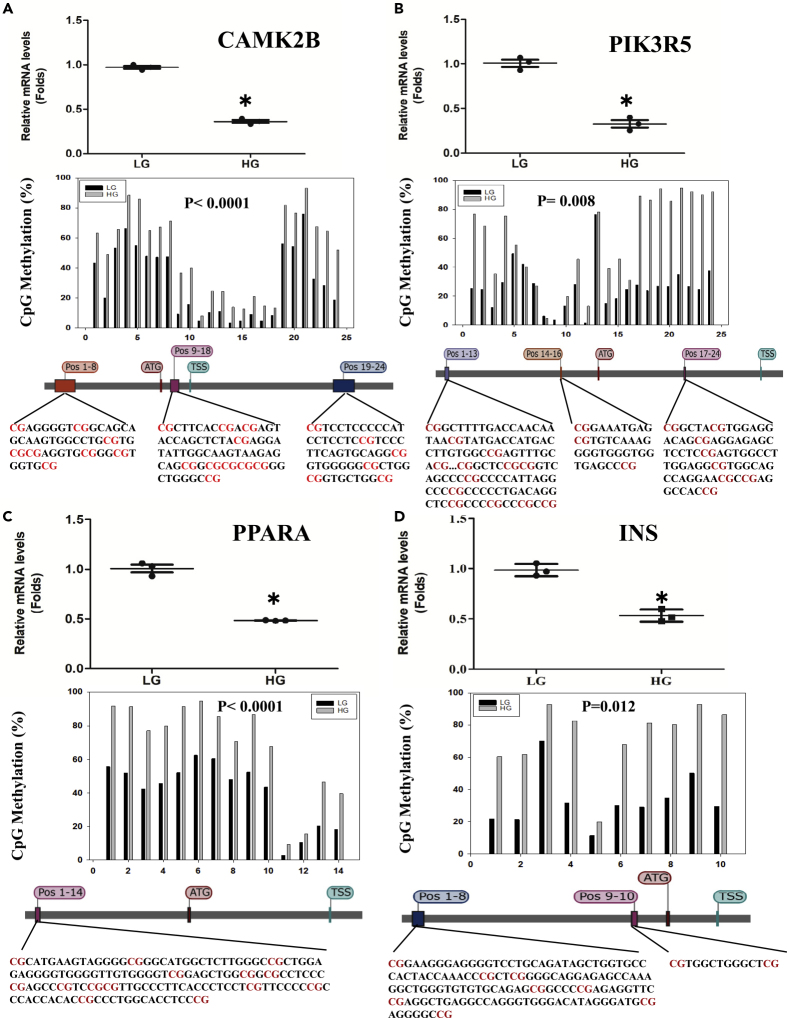
The Relationship between DNA CpG Methylation and Gene Expression (A–D) Huh-7 cells were cultured in HG or LG media for 72 h, and the methylation levels of the CpG sites of key genes involved in signaling pathways were analyzed by tNGBS. Huh-7 cells were cultured in HG media for 12 h, and the mRNA levels of these genes were determined by real-time RT-PCR analysis. The CpG methylation of the CAMK2B gene is shown in (A), where the top image shows the mRNA levels, the middle shows the methylation levels of 5′ upstream (Pos 1–14) region, and the bottom shows the sequence. CpG methylation of the PIK3R5 gene is shown in (B), where the top image shows the mRNA levels; the middle shows the methylation levels of 5′ upstream (Pos 1–13), intron (Pos 14–16), and exon (Pos 17–24) regions; and the bottom shows the gene sequence. CpG methylation of the PPARα gene is shown in (C), where the top shows the mRNA levels; the middle, the methylation levels of 5′ upstream (Pos 1–8), exon (Pos 9–12), and intron (Pos 13–24) regions; and the bottom, the gene sequence. CpG methylation of the INS gene is shown in (D), where the top image shows the mRNA levels; the middle, the methylation levels of 5′ UTR (Pos 1–2) and 5′ upstream (Pos 3–10) regions; and the bottom, the gene sequence. Data are represented as mean ± SD and statistic by t test. ∗Significant difference in expression between LG and HG (p < 0.05).

To confirm the results from WGBS, the methylation levels of CpG sites in promoter regions of PPARα, INS, CAMK2B, and PIK3R5, key genes in NAFLD, type II diabetes mellitus, insulin secretion, and cAMP signaling pathways, were determined using target next-generation bisulfite sequencing (tNGBS) (Figure 5). The results showed that HG increases the methylation levels of every CpG site not only in promoter region but also in exon and intron regions. For CAMK2B gene (Figure 5A), 24 CpG sites were identified including 8 (Pos 1–8) in promoter region, 4 (Pos 9–12) in exon region, and 12 (Pos 13–24) in intron region. HG increases the methylation levels of Pos 1–8 by 22%, Pos 9–12 by 18%, and Pos 13–24 by 19%. For PIK3R5 gene (Figure 5B), 24 CpG sites were identified including 8 (Pos1–8) in exon region, 13 (Pos 9–21) in promoter region, and 3 (Pos 22–24) in intron region. HG increases the methylation level of Pos 1–8 by 60% on average, Pos 9–21 by 15%, and Pos 22–24 by 19%. For PPARα gene (Figure 5C), HG increases the methylation levels of 14 CpG sites (Pos 1–14) in promoter region by 27%. For INS gene (Figure 5D), HG increases the methylation levels of 10 CpG sites (Pos 1–10) by 40% on average.

To explore whether DNA methylation in promoter region is involved in down-regulation of gene expression, four multiple function genes were selected for confirmation (Figure 5). Gene expressions were estimated by RT-PCR, and CpG methylations were confirmed by tNGBS. The relationship of gene expression with CpG methylation of CAMK2B is shown in Figures 5A; with PIK3R5, in 5B; with PPARα, in 5C; and with INS, in 5D. The results showed that all these gene expressions were significantly decreased when Huh-7 cells were cultured in HG, which is highly consistent with the results from the tNGBS analysis. The results confirm that the CpG methylation in the genes is responsible for the down-regulation of their expression.

## Discussion

This study proposes a regulatory pathway for HG to lead to induction of lipid accumulation through 25HC and epigenetic regulation through hypermethylation. HG induces lipid accumulation through increased nuclear 25HC levels. The potent regulatory molecule 25HC, but not 27HC, activates DNMT1, which methylates cytosine of CpG in promoter regions, suppresses their expression, and down-regulates the associated signaling pathways. This proposed mechanism sheds new light on understanding how consuming excess sugar can lead to an increase in lipid biosynthesis, lipid accumulation, and risk of weight gain. These results propose some of the epigenetic mechanisms by which high-sugar diet may lead to insulin resistance and inflammation, both of which are risk factors for metabolic syndrome, through epigenetic regulation.

Sugars, such as fructose and glucose, can be catabolized to acetyl-CoA, which is the major substrate for producing energy, ATP, *in vivo*. When ATP concentrations reach high levels, excess acetyl-CoA is shunted into synthesis of cholesterol, free fatty acids, and triglycerides as a means of energy storage. The correlation between cholesterol and triglyceride biosynthesis has not been elucidated. In the present report, we demonstrate evidence that HG increases nuclear 25HC levels and subsequently increases triglyceride biosynthesis. We also may have explored for the first time that 25HC serves as an activator of DNMT1 and plays an important role in global regulation of metabolic pathways, cell survivals, and cell death by regulating critical signaling pathways. It has been reported that 25HC as a ligand of liver X receptor (LXRs) plays an important role in the control of lipid metabolism (Janowski et al., 1996). LXR ligands up-regulate expression of cholesterol reverse transporters such as ABCA1 and ABCG1 via activation of LXR/RXR heterodimers. ABCA1/G1 is known to mediate efflux of cellular cholesterol and phospholipids, which is the target for therapy of anti-atherosclerosis (Zhu et al., 2012). Unfortunately, administration of the synthetic ligands to mice triggers induction of the lipogenic pathway and elevates plasma triglyceride levels (Schultz et al., 2000). Addition of 25HC to human hepatocytes increases gene expression of key enzymes, ACC and FAS, in lipid biosynthesis and increases intracellular lipids levels via SREBP-1C pathway (Ma et al., 2008). 25HC has also been described to function in immune system, suppressing immunoglobulin production in B cells (Bauman et al., 2009) and inducing expression of the inflammatory cytokine interleukin-8 (Wang et al., 2004). Recently, 25HC has been shown to directly restrict target cell entry of enveloped viruses by inhibiting fusion of virus and cell membranes (Doms et al., 2018, Ke et al., 2017, Li et al., 2017, Liu et al., 2013, Wang et al., 2017). More interestingly, it has been reported that 25HC can be sulfated to 25-hydroxycholesterol 3-sulfate, 25HC3S, which has been shown as a distinct yet potent regulator of cellular functions (Li et al., 2007, Ren et al., 2007). Both 25HC3S and 25HC play important roles in lipid metabolism, inflammatory responses, and cell survival but act in a direction opposite to each other (Ren and Ning, 2014). The observation of global regulation is consistent with the present conclusion that 25HC serves as an agonist of epigenetic regulator, DNMT1. Whether the sulfation of 25HC to 25HC3S is the fundamental regulatory mechanism needs to be further investigated.

It is also interesting to notice that HG hyper-methylates CpG but hypomethylates CHG and CHH in whole gene regions including CpG island, CpG island shore, promoter, and TSS region as shown in Figure 4C. It is well-believed that CpG methylation in promoter regions is directly related with silencing gene expression (Lewis et al., 1992). However, the physiological significance of hypomethylation of CHG and CHH is unknown. DNA methylation is established and maintained by three essential DNA methyltransferases, DNMT1, DNMT3a, and DNM3b, in mammals (Koerner and Barlow, 2010, Long et al., 2017). Methylated DNA is then recognized by methyl CpG-binding proteins along with associated co-repressors that lead to silencing of the associated promoter (Clouaire and Stancheva, 2008). The *in vivo* role of DNMT1 in carcinogenesis has been widely studied using hypomorphic mice instead of DNMT1−/− mice that die during embryogenesis (Subramaniam et al., 2014). DNMT1 hypomorphic mice exhibit resistance to alcohol-induced hepatic steatosis compared with the wild-type mice (Kutay et al., 2012). It has been reported that metabolic syndrome has an impact on the global methylation pattern in visceral adipose tissue (Castellano-Castillo and Moreno-Indias, 2019). The results indicate that the steatosis is most likely dependent on the DNA methylation in related genes, which silences gene expression. In this study, we report that HG incubation induces DNA CpG methylation in promoter regions of the key signaling pathways, which induces lipid accumulation in human hepatocytes. We found that HG incubation increases 25HC levels in hepatocyte nuclei. 25HC activates DNMT1 and increases ^5m^CpG in promoter regions of 2,225 genes.

Furthermore, the HG-induced CpG methylation in promoter regions covers 79 KEGG pathways. Fifty-seven pathways were identified as significantly hypermethylated. To our knowledge, 13 of these pathways are directly related to lipid metabolism, including metabolic signaling pathway, Ras signaling pathway (Ye et al., 2017), cAMP signaling pathway (Ravnskjaer et al., 2016), PI3K-Akt signaling pathway (Zhou et al., 2019), MAPK signaling pathway (Yang et al., 2018), Wnt signaling pathway (Yao et al., 2018), NAFLD (Engin, 2017), type 1 diabetes mellitus (Ritchie et al., 2017), cGMP-PKG signaling pathway (Schwarz et al., 2018), FoxO signaling pathway (Cheng and White, 2011), type II diabetes mellitus (Samuel and Shulman, 2012), insulin secretion (Eto et al., 2002), and insulin signaling pathway (Consitt et al., 2009) as shown in Table 1. As hypermethylation in promoter regions is believed to silence the gene expression, it is reasonable to conclude that HG induces metabolic disorders via methylation of their key genes in the pathways, which were confirmed by analysis of mRNA levels as shown in Figure 5. Only one (p < 0.05) tight junction pathway was identified as hypomethylation. However, its physiological significance is unknown.

**Figure 6:**
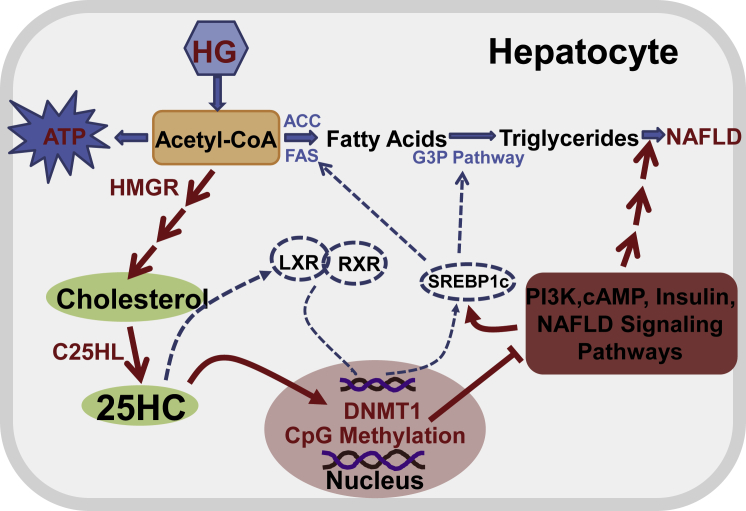
Proposed Regulatory Pathway of Intracellular Lipid Homeostasis A high-sugar diet produces an excess of acetyl-CoA, which is the energy source for generation of ATP. The excess of acetyl-CoA can also be used for synthesis of cholesterol and subsequently for synthesis of oxysterols such as 25-hydroxycholesterol by key enzymes, 3-hydroxy-3-methyl-glutaryl-CoA reductase and cholesterol 25-hydroxylase (C25HL) (Lund et al., 1998). 25HC activates LXR and upregulates SREBP-1C expression, which results in increasing biosynthesis of free fatty acids and triglycerides in a known pathway. On the other hand, 25HC enters nuclei and activates DNMT1, which selectively methylates CpG in promoter regions and silences gene expression, leading to down-regulation of many signaling pathways including PI3K, cAMP, insulin, NAFLD, and metabolic pathways. The dashed blue lines represent known pathways, and the red solid lines represent the proposed pathways. The proposed pathways play an important role in the occurrence of NAFLD and metabolic syndrome.

The current results most likely represent the etiology for the development of high sugar diet-induced fatty liver diseases. Based on the results, we summarize the regulatory pathway, which may play an important role in the occurrence of NAFLD and metabolic syndrome. When cells are incubated in HG media, high sugar consumption will increase intracellular 25HC, which in turn will activate DNMT1, as shown in Figure 6.

### Limitations of the Study

We are investigating the effect of knockout and knockdown of 25HC syntheses on the activity of DNA methyltransferases and considering this as a limitation of this study. The enzyme kinetic results provide strong advance of this question. All the materials are available to all interested scientists per request. Subsection lead contact to Yaping Wang.

## Methods

All methods can be found in the accompanying Transparent Methods supplemental file.

## Supplemental Information

Document S1. Transparent Methods and Tables S1, S3, and S4

Table S2. Lipidomic Results of Huh-7 Cells Cultured in LG and HG Media, Normal Human Liver Tissue and that of Patients with NASH, and 25HC and 27HC Standard, Related to Figure 1

Table S5. Significant Enrichment KEGG Pathways of DMGs in Promoter Regions under CG, CHG, and CHH Contexts, Related to Figure 4
