# Supplementary material for: High Glucose Induces Lipid Accumulation via 25-Hydroxycholesterol DNA-CpG Methylation
Source: iScience. 2020 Apr 29;23(5):101102. doi: 10.1016/j.isci.2020.101102 (PMC7225732; doi:10.1016/j.isci.2020.101102)
Supplement: Document S1. Transparent Methods and Tables S1, S3, and S4 [file mmc1.pdf]

iScience, Volume *23*

Supplemental Information

High Glucose Induces Lipid Accumulation via 25-Hydroxycholesterol DNA-CpG Methylation

Yaping Wang, Lanming Chen, William M. Pandak, Douglas Heuman, Phillip B. Hylemon, and Shunlin Ren

SUPPLEMENTAL INFORMATION

Table S 1. Primer Sequence for Real-time Polymerase Chain Reaction, Related to Figure 1, Figure 3 and Figure 5.

Gene name	Forward primer	Reverse primer
SREBP1	CAGCCCCACTTCATCAAGG	ACTGTTGCCAAGATCAAGG
DNMT1	TACCTGGACGACCCTGACCTC	CGTTGGCATCAAAGATGGACA
INS	CTATCCAGCGTACTCCAAAG	ACAAGTCTGAATGCTCCACT
CAMK2B	GTCCACCGCGGCCTC	TTTTGGTGCTATTCGTCTGGG
PPARA	TCGACTCAAGCTGGTG	TTCCTGAGAGGATGACCC
PIK3R5	TCGCAAGGACGAGGGATCCTC	GTCTTCATATTTGGGTCGTTATG
β-actin	CATGTACGTTGCTATCCAGGC	CTCCTTAATGTCACGCACGAT
GAPDH	CAATGACCCCTTCATTGACC	TTGATTTTGGAGGGATCTCG

Table S 3. Fold Change of Nuclear Lipid Levels Between HG and LG Cultured Huh-7 Cells, Related to Figure 1.

Lipid name	Folds	Lipid name	Folds
Cer (d26:1)	53.44	PE (18:1e_22:4)	4.75
Cer (d18:2_18:0)	7	PE (18:1_22:6)	5.08
Cer (m18:1_23:0)	4.95	PG (38:1e)	6.24
ChE ()	55.57	PI (16:0_18:1)	199.62
DG (14:0_18:2)	63.45	PI (18:0_18:2)	40.53
DG (16:0_18:2)	5.01	PIP (42:9e)	10.35
DG (18:3_18:2)	6.05	PS (16:0_18:2)	20.32
Hex1Cer (d18:1_16:0)	21.52	PS (16:0_20:4)	10.97
Hex1Cer (d18:1_24:1)	250.23	PS (18:0_18:2)	29.64
LPE (18:1)	6.59	SM (d34:2)	19.37
LPE (22:6)	5660.37	TG (16:0_9:0_18:2)	10.26
PC (30:0)	17.06	TG (16:1_13:0_14:0)	10.93
PC (16:1_16:1)	12.05	TG (16:1_10:0_18:1)	31.37
PC (37:3e)	6.23	TG (10:0_18:2_18:2)	57.11
PC (18:1_20:4)	5.33	TG (16:0_14:4_18:3)	41.59
PC (38:5)	7.67	TG (16:1_14:1_18:2)	15.98
PC (18:0_20:2)	22.1	TG (16:0_18:1_18:1)	19.06
PC (38:1)	12.73	TG (18:1_18:1_18:1)	49.69
PC (40:5)	4.51	TG (16:0_20:2_20:4)	12.23
PE (16:0p_18:1)	18.93	TG (18:0_18:1_20:5)	49.7
PE (18:0_16:0)	5.41	SM (d41:2)	5.54
PE (18:1_18:2)	5.58	SPH (d18:1)	2.15
PE (16:0_20:3)	5.31	ZyE ()	87.39
PE (18:1_18:1)	81.3	**25HC**	**0.37**
PE (18:1e_20:4)	8.49	**27HC**	**-0.91**
PE (18:1e_22:5)	94.3		

Table S 4. Fold Change of Nuclear Lipid Levels Between Human NASH and Normal Liver Tissues, Related to Figure 1

Lipid name	Folds	Lipid name	Folds
AEA (15:1)	3.55	PG (18:1_18:2)	5.8
CL (18:1_16:1_16:1_18:1)	2.75	PG (28:1_16:0)	102.71
CL (20:4_16:0_18:0_18:1)	9.46	PG (38:1e)	80.39
Cer (t20:0_23:3)	2.11	PG (18:1_18:1)	3.94
Cer (d18:1_23:3)	2.54	PG (18:1_18:2)	2.26
Cer (m18:0_24:0)	17.35	PS (18:0_22:5)	2.09
ChE ()	7.66	SM (d40:1)	2.73
ChE ()	42.3	SM (d41:1)	2.29
CmE (30:6)	2.8	SM (d42:3)	5.61
CmE (20:4)	2.03	TG (16:0_18:1_18:1)	6.16
CmE (10:0)	15.94	TG (16:0_18:1_18:2)	8.15
Co (Q10)	2.42	TG (16:0_18:1_18:3)	32.11
Co (Q10)	9.25	TG (18:1_18:1_18:1)	10.35
Co (Q9)	2.34	TG (16:1_16:1_18:1)	29.59
DG (16:0_18:1)	6.9	TG (18:1_18:1_18:2)	14.48
DG (36:4e)	6.15	TG (16:0_16:1_18:1)	4.22
DG (18:1_18:1)	6.94	TG (16:0_16:0_18:1)	2.68
DG (18:1_18:2)	12.46	TG (16:0_14:0_18:2)	38.08
DG (16:0_18:2)	9.1	TG (18:1_18:2_18:2)	40.05
DG (32:1e)	10.05	TG (16:0_18:2_18:3)	72.33
DG (34:2e)	12.14	TG (16:0_18:1_18:2)	216.21
DG (16:0_16:0)	2.61	TG (18:1_14:0_18:3)	85.11
DG (16:1_18:2)	15.2	StE (4:0)	2.45
DG (34:3e)	15.38	StE (30:5)	5.05
Hex1Cer (d18:1_24:1)	2.65	ZyE (13:1)	2.08
LPE (18:1)	3.81	ZyE ()	4.23
MLCL (18:2_18:2_18:2)	2.69	**25HC**	**0.31**
PG (28:0_16:0)	50.44	**27HC**	**0.004**
PG (28:0_16:0)	480.71		

TRANSPARENT METHODS

Materials

Cell culture reagents and supplies were purchased from GIBCO BRL (Grand Island, NY); 25HC was purchased from New England Nuclear (Boston, MA). Huh-7 cells were obtained from American Type Culture Collection (Rockville, MD). Human liver tissues were purchased from Sekisui XenoTech, LLC (Kansascity, KS). The reagents for real time RT-PCR were from AB Applied Biosystems (Warrington, UK). The chemicals used in this research were obtained from Sigma Chemical Co. (St. Louis, MO) or Bio-Rad Laboratories (Hercules, CA). Polyclonal mouse antibodies against SREBP-1C, were purchased from Santa Cruz Biotechnology (Santa Cruz, CA), and DNMT1 was purchased from AbcamInc. (Burlingame, CA). All solvents were obtained from Fisher (Fair Lawn, NJ) unless otherwise indicated. Biochemical assay reagents were purchased from Wako Chemicals USA, Inc. (Richmond, VA). For lipidomic analysis, internal standards were purchased from Avanti Polar Lipids (Alabaster, AL) as their premixed splash lipidomixmass spec standard. Internal standards were added to samples in 10 μL aliquots. Standards included 15:0-18:1(d7) PC, 15:0-18:1(d7) PE, 15:0-18:1(d7) PS, 15:0-18:1(d7) PG, 15:0-18:1(d7) PI, 15:0-18:1(d7) PA, 18:1(d7) LPC, 18:1(d7) LPE, 18:1(d7) Cholesterol Ester, 18:1(d7) MAG, 15:0-18:1(d7) DAG, 15:0-18:1(d7)-15:0 TAG, 18:1(d9) SM, and Cholesterol (d7).For LC-MS/MS analyses, a Thermo Scientific Q Exactive HF Hybrid Quadrupole-Orbitrap Mass Spectrometer was used. Samples were separated via a Thermo Scientific Vanquish Horizons UHPLC System functioning in binary mode.

Cell Culture

Huh-7 cells were cultured in DMEM medium supplemented with 10% heat-inactivated fetal bovine serum (FBS), with either low glucose (LG, 1.5 g/L) or high glucose (HG, 4.5 g/L) at 37 ℃ in a humidified atmosphere of 5% CO_2.

Qualification and Quantification of Intracellular Neutral Lipids in Hepatocytes

Oil Red O Staining: Huh-7 cells were seeded on 22 × 22 mm glass coverslips in six-well plates. Cells were cultured in DMEM media with LG or HG for 0, 36 and 72 hrs, fixed with 3.7% formaldehyde in PBS (phosphate-buffered saline) for 30 min, followed by three washes with PBS. The cells were stained with 0.2% Oil Red O in 60% isopropanol for 10 min and washed three times with PBS. Cell nuclei were stained with Harris hematoxylin for 1 minute, washed with PBS three times. The stained lipids were examined by microscopy (Ma et al., 2008).

Total levels of cellular triglyceride (TG), total cholesterol, cholesterol ester, and free fatty acids were determined by biochemical kits according to the manufacturer's instructions. Lipids contents were normalized to protein concentrations tested with protein quantitative assay kit (Bio-Rad) (Xu et al., 2013). Briefly, Huh-7 cells were homogenized, and then lipids were extracted with a mixture of chloroform and methanol (2:1, vol/vol). The filtered extracts, 0.2 ml, were evaporated to dryness and dissolved in 100 μl of isopropanol containing 10% of triton X-100 for total cholesterol and cholesterol esters assay (Wako Chemicals USA, Richmond, VA); NEFA solution (0.5 g of EDTA-Na_2, 2 g of Triton X-100, 0.76 ml of 1N NaOH, and 0.5 g of sodium azide/l, pH 6.5), for free fatty acid assay (Wako Chemicals USA, Richmond, VA); and isopropanol only, for triglyceride assay (Fisher Scientific, Pittsburgh, PA), respectively.

Extraction and Determination of DNA and mRNA Levels

Genomic DNA from 5,000 Huh-7 cells cultured in DMEM medium with LG or HG for 72 hours were extracted using QIAamp DNA Mini Kit (QIAGEN, Hilden, Germany). Each sample, 2 μg, was sent to EpigenDx, Inc. (Hopkinton, MA) for analysis of global methylation and target next generation bisulfite sequencing. The same samples, 6 μg /each, were sent to Novogene Co., Ltd (Tianjin, China) for analysis of whole genome bisulfite sequencing (WGBS). Total RNA was isolated with an SV total RNA isolation system (Promega, Madison, WI) with DNase treatment. Each sample, 2 μg, was used for the first-strand cDNA synthesis as recommended by the

manufacturer (Invitrogen, Carlsbad, CA). Real-time RT-PCR was performed using SYBR Green as the indicator on ABI 7500 Fast Real-Time PCR System (Applied Biosystems, Foster City, CA). Amplifications of β-actin or GAPDH were used as internal controls. Relative messenger RNA (mRNA) expression was quantified with the comparative cycle threshold (Ct) method using the primer set shown in **Table S1** and was expressed as $2^{-\Delta\Delta Ct}$ (Livak and Schmittgen, 2001).

Western blot Analysis

Specific proteins in nuclei were analyzed by western blot. The nuclear proteins were extracted using NE-PER Nuclear and Cytoplasmic Extraction Reagents (Fisher Scientific). For each sample, 10 μg of nuclear proteins were separated on 8%-12% SDS polyacrylamide gel electrophoresis (SDS-PAGE) gels. Electrophoresis was performed at 100V for 15 min and 200V for another 25 min in a Bio-Rad mini-gel system. After electrophoresis, samples were transferred onto a polyvinylidene difluoride (PVDF) membrane at 30V for 50 min. The specific proteins on the membrane were detected by incubation with various primary antibodies including anti-SREBP-1C (Santa Cruz Biotechnologies) oranti-DNMT1 (Abcam) at 4°C overnight, followed by further incubation with an appropriate horseradish peroxidase (HRP)-conjugated secondary antibody (Bio-Rad) at room temperature for 1 hr. Lamin B1 protein levels were used as controls for equal nuclear protein loading. Each positive band was quantified by Advanced Image Data Analyzer (Aida, Straubenhardt, Germany) (Zhang et al., 2012).

Lipidomic Analysis of Nuclear Neutral Lipids

Extraction of Nuclear Lipids

The human liver tissue (80 mg) from normal people or NASH patients were homogenized in 1 ml PBS and centrifuged. Huh-7 cells were cultured in 100 mm dishes with 8 ml of media with LG or HG for 72 hrs. The cells were washed twice with cold phosphate-buffered saline (PBS). Both liver and Huh-7 cellular pellets were resuspended in 0.5 ml cytoplasmic extraction reagent I and incubated on ice for 10 min. Then 27.5 μl of

cytoplasmic extraction reagent II were added and the mixture was incubated on ice for 1 min, centrifuged for 5 min at 16,000 × g, the supernatant was completely removed, and the pellet was washed twice with cold PBS. Fifty µl of nuclear extraction reagent, 450 µl of PBS and 50 µl of proteinase K (1mg/ml) were added to the tube and the mixture was incubated for 3 hrs at 50 °C. Total lipids were extracted and partitioned by adding 3.3 volumes of chloroform: methanol (1:1) as previously described (Ren et al., 2006). Cholesterol and hydroxycholesterols were distributed into the chloroform phase.

The extracted lipids in chloroform phases from both human liver tissues and Huh-7 cells were collected into 13 x 100 mm borosilicate tubes with a Teflon-lined cap (West Chester, PA), respectively. Then 2 mL of CH_3OH and 1 mL of $CHCl_3$ were added along with the internal standard cocktail (10 µL). The contents were dispersed using an ultra sonicator at room temperature for 30 s. This single phase mixture was incubated at 48 °C overnight. Debris was then pelleted in a centrifuge for 5 minutes at 5000 × g, and the supernatant was transferred to a clean tube. The extract was reduced to dryness using a Speed Vac. The dried residue was reconstituted in 0.2 ml of the starting mobile phase solvent for untargeted analysis, sonicated for 15 sec, then centrifuged for 5 minutes in a tabletop centrifuge before transfer of the clear supernatant to the auto-injector vial for analysis.

Untargeted analysis

The lipids were separated by reverse phase LC using a Thermo Scientific Accucore Vanquish C18+ 2.1 (i.d.) x 150 mm column with 1.5 µm particles. The HPLC used a binary solvent system at a flow rate of 0.26 mL/min with a column oven set to 55 ℃. Prior to injection of the sample, the column was equilibrated for 2 min with a solvent mixture of 99% Mobile phase A1 (CH_3CN/H_2O, 50/50, v/v, with 5 mM ammonium format and 0.1% formic acid) and 1% Mobile phase B1 ($CH_3CHOHCH_3/CH_3CN/H_2O$, 88/10/2, v/v/v, with 5 mM ammonium format and 0.1% formic acid) and after sample injection (typically 10 µL), the A1/B1 ratio was maintained at 99/1 for 1.0 min, followed by a linear gradient to 35% B1 over 2.0 min, then a linear gradient to 60% B1 over 6 min,

followed by a linear gradient to 100% B1 over 11 min., which held at 100% B1 for 5 min, followed by a 2.0 min gradient return to 99/1 A1/B1. The column was re-equilibrated with 99:1 A1/B1 for 2.0 min before the next run. Each sample was injected two times for analysis in both positive and negative modes. For initial full scan MS (range 300 to 200 *m/z*) the resolution was set to 120,000 with a data-dependent MS^2 triggered for any analyte reaching 3E6 or above signal. Data-dependent MS^2 were collected at 30,000 resolution. Data was analysed using Thermo Scientific's Lipid Search 4.2 software.

Analysis of Cholesterol and Oxysterol Biosynthesis

After incubation of Huh-7 cells in 100 mm dishes with 8 ml of media with LG or HG for 72 hrs, 5 µCi of [1-^{14}C]-acetate was added and culturing was continued for another 3 or 9 hrs. After 9 hrs of incubation at 37 ℃, the media was removed and the cells were washed twice with cold PBS, harvested in 1ml PBS, and collected in microcentrifuge tubes. The cells were sedimented by centrifugation, and the pellets were washed three times by resuspension and sedimentation. The nuclear lipids were extracted as described above. [^{14}C]-Acetate derivatives in the chloroform phase were analyzed by HPLC on a Waters Spherisorb Analytical C18 column (5 µm, 4.0 mm x 50 mm). The mobile phase consisted of (A), 5 mM ammonia acetate in 95% of water, 5% acetonitrile, and (B), 5 mM ammonia acetate in 95% of methanol, 5% of acetonitrile. The 45 min gradient was as follows: 0-25.0 min, 60%-100% B linear; 25.0-40.0 min, 100% B; 40.0-45.0 min, 100%-60% B linear. The elution stream from the HPLC column was monitored by OD_{202mm} using Agilent Series 1100 solvent delivery system (Hewlett Packard) at 0.5 ml/min flow rate. The eluted fractions were collected every 0.5 min (0.25 ml per fraction) except as indicated. The counts of [^{14}C]-acetate derivatives in each fraction were determined by Scintillation Counting. The column was calibrated with cholesterol, 25-hydroxycholesterol, and 27-hydroxycholesterol.

Enzyme Kinetic Study of 25-Hydroxycholesterol as Epigenetic Regulator

The activities of DNMT1, DNMT3a, DNMT3b, GCN3 (Giant congenital nevi), p300 (histone acetyl

transferase), Pcaf (KAT2B lysine acetyltransferase 2B), HDAC1 (histone deacetylase 1), HDAC2 (histone deacetylase 2), HDAC3 (histone deacetylase 3), HDAC6 (histone deacetylase 6), HDAC10 (histone deacetylase 10), and KDM6B-JMJD3 (lysine demethylase 6B) were measured as instructed by reaction biology company (Reaction Biology Corp. PA, USA).

For the DNMT1 activity assay, the substrate solution, 0.001 mg/ml Poly (dI-dC):Poly (dI-dC)in 50 mM Tris-HCl, pH 7.5, 50 mM NaCl, 5 mM EDTA, 5 mM DTT, 1 mM PMSF, 5% glycerol, 0.01% Brij35, 1% DMSO, was used. For the DNMT3a/3b activity assay, 0.0075 mg/ml Lambda DNA in 50 mM Tris-HCl, pH 7.5, 50 mM NaCl, 5 mM EDTA, 5 mM DTT, 1 mM PMSF, 5% glycerol, 1% DMSO, was used. The indicated DNMT1, DNMT3a and DNMT3b was added to the appropriate substrate solution and gently mixed, amounts of cholesterol, 25HC or 27HC ranging from 5.08E-09 to 0.0001 M in DMSO were added to the reaction mixture by using Acoustic Technology (Echo 550, LabCyte Inc. Sunnyvale, CA). The mixtures were incubated for 15 min, ^{3}H-SAM was added to the reaction mixture to initiate the reaction, and the mixture was incubated for 60 min at 30℃. Following incubation, the reaction mixture was transferred to filter-paper for detection of radioactivity counts.

Analysis of Global Methylation, Long Interspersed Nucleotide Element 1(LINE-1) Assay

For global DNA methylation analysis, 500 ng of extracted genomic DNA was bisulfite-treated using the EZ DNA Methylation kit (Zymo Research, Inc., CA). PCR reaction and product purification were performed as per the manufacturer's protocol (GE Healthcare Life Sciences).Ten μL of the PCR products were sequenced by Pyrosequencing on the PSQ96 HS System following the manufacturer's instructions (Pyrosequencing, Qiagen). The methylation status of each CpG site was determined individually as an artificial C/T SNP using QCpG software (Pyrosequencing, Qiagen). The methylation level at each CpG site was calculated as the percentage of the methylated alleles divided by the sum of all methylated and unmethylated alleles. The mean methylation level was calculated using methylation levels of all measured CpG sites within the targeted region of each gene. Each

experiment included non-CpG cytosines as internal controls to detect incomplete bisulfite conversion of the input DNA. In addition, a series of unmethylated and methylated DNA were included as controls in each PCR. Furthermore, PCR bias testing was performed by mixing unmethylated control DNA with in vitro methylated DNA at different ratios (0%, 5%, 10%, 25%, 50%, 75%, and 100%), followed by bisulfite modification, PCR, and Pyrosequencing analysis.

Analysis of Whole Human Genome Bisulfite Sequencing (WGBS)

Each sample, 5.2 μg, of genomic DNA spiked with 26 ng lambda DNA was fragmented by sonication to 200-300 bp with Covaris S220, followed by end repair and adenylation. Cytosine-methylated barcodes were ligated tosonicated DNA per manufacturer's instructions. These DNA fragments were treated twice with bisulfiteusing EZ DNA Methylation-Gold TM Kit (Zymo Research), before the resulting single-strand DNA fragments were PCR amplificated using KAPA HiFi Hot Start Uracil and Ready Mix (2X). Library concentration was quantified by Qubit® 2.0 Flurometer (Life Technologies, CA, USA) and quantitative PCR, and the insert size was assayedon an Agilent Bioanalyzer 2100 system.

The library preparations were sequenced on an Illumina Hiseq 2500/4000 or Novaseq platform and 125 bp/150 bp paired-end reads were generated. Image analysis and base calling were performed with Illumina CASAVA pipeline, and finally 125 bp/150 bp paired-end reads were generated. Trimmomatic (Trimmomatic-0.36) software was used forquality control. Bismark software (version 0.16.3; Krueger F, 2011) was used to perform alignments of bisulfite-treated reads to a reference genome (-X 700 --dovetail). DSS software (Park and Wu, 2016) was used to identify differentially methylated regions (DMRs). KOBAS software (Mao et al., 2005) was used to test the statistical enrichment of DMR related genes in the Kyoto Encyclopedia of Genes and Genomes (KEGG) pathways.

Target Next Generation Bisulfite Sequencing

The samples were prepared as described above for the LINE-1 assay. Libraries were prepared using a custom Library Preparation method created by EpigenDx. Next, library molecules were purified using AgencourtAMPure XP beads (Beckman Coulter) and quantified using the Qiagen QIAxcel Advanced System. Barcoded samples were then pooled in an equimolar fashion before template preparation and enrichment were performed on the Ion Chef™ system (Thermo Fisher) using Ion 520™ & Ion 530™ ExT Chef reagents. Following this, enriched, template-positive library molecules were then sequenced on the Ion S5™ sequencer using an Ion 530™ sequencing chip (Thermo Fisher). FASTQ files from the Ion Torrent S5 server were aligned to the local reference database using open-source Bismark Bisulfite Read Mapper with the Bowtie 2 alignment algorithm. Methylation levels were calculated in Bismark by dividing the number of methylated reads by the total number of reads.

SUPPLEMENTAL REFERENCE

Livak, K.J., and Schmittgen, T.D. (2001). Analysis of relative gene expression data using real-time quantitative PCR and the 2(-Delta Delta C(T)) Method. Methods (San Diego, Calif) *25*, 402-408.

Ma, Y., Xu, L., Rodriguez-Agudo, D., Li, X., Heuman, D.M., Hylemon, P.B., Pandak, W.M., and Ren, S. (2008). 25-Hydroxycholesterol-3-sulfate regulates macrophage lipid metabolism via the LXR/SREBP-1 signaling pathway. American journal of physiology Endocrinology and metabolism *295*, E1369-1379.

Mao, X., Cai, T., Olyarchuk, J.G., and Wei, L. (2005). Automated genome annotation and pathway identification using the KEGG Orthology (KO) as a controlled vocabulary. Bioinformatics (Oxford, England) *21*, 3787-3793.

Park, Y., and Wu, H. (2016). Differential methylation analysis for BS-seq data under general experimental design. Bioinformatics (Oxford, England) *32*, 1446-1453.

Ren, S., Hylemon, P., Zhang, Z.P., Rodriguez-Agudo, D., Marques, D., Li, X., Zhou, H., Gil, G., and Pandak, W.M. (2006). Identification of a novel sulfonated oxysterol, 5-cholesten-3beta,25-diol 3-sulfonate, in hepatocyte nuclei and mitochondria. Journal of lipid research *47*, 1081-1090.

Xu, L., Kim, J.K., Bai, Q., Zhang, X., Kakiyama, G., Min, H.K., Sanyal, A.J., Pandak, W.M., and Ren, S. (2013). 5-cholesten-3beta,25-diol 3-sulfate decreases lipid accumulation in diet-induced nonalcoholic fatty liver disease mouse model. Molecular pharmacology *83*, 648-658.

Zhang, X., Bai, Q., Kakiyama, G., Xu, L., Kim, J.K., Pandak, W.M., Jr., and Ren, S. (2012). Cholesterol metabolite, 5-cholesten-3beta-25-diol-3-sulfate, promotes hepatic proliferation in mice. The Journal of steroid biochemistry and molecular biology *132*, 262-270.
